# Supplementary material for: Effective antibiotic dosing in the presence of resistant strains
Source: PLoS One. 2022 Oct 10;17(10):e0275762. doi: 10.1371/journal.pone.0275762 (PMC9551627; doi:10.1371/journal.pone.0275762)
Supplement: S1 Appendix — (PDF) [file pone.0275762.s001.pdf]

A Appendix

A.1 Proof of Theorem 3.1

Suppose

$$\Omega_5 \quad = \quad \{(S, A, x, u, v)|S, A, x, u, v \geq 0\}$$

In Ω_5, the following inequalities hold

$$\begin{aligned} S' &= d_s S^0 \geq 0 \quad \text{when } S = 0 \text{ and } A, x, u, v \geq 0 \\ A' &= d_A A_0(t) \geq 0 \quad \text{when} \quad A = 0 \text{ and } S, x, u, v \geq 0 \\ x' &= K_2(S, A)v \geq 0 \quad \text{when} \quad x = 0 \text{ and } S, A, u, v \geq 0 \\ u' &\geq 0 \quad \text{when} \quad u = 0 \text{ and } S, A, x, v \geq 0 \\ v' &= k_4 xu \geq 0 \quad \text{when} \quad v = 0 \text{ and } S, A, x, u \geq 0 \end{aligned}$$

This implies that the non-negative Ω_5 in $\mathbb{R}^5$ is positively invariant for the model (3). For the second part of the theorem, consider

$$\begin{aligned} \gamma S' + x' + u' + v' &= \gamma d_s(S^0 - S) - d_x x - \alpha xu - d_u - K_1(S, A)u - k_3 xu - d_+ v - k_4 xu \\ &\leq \gamma d_s S^0 - d_{min}(\gamma S + x + u + v) \end{aligned}$$

where $d_{min} = \min\{d_s, d_x, d_u, d_+\}$. Assuming $Y = \gamma S + x + u + v$, then

$$Y' \leq \gamma d_s S^0 - d_{min} Y$$

The inequality takes the form

$$Y(t) \leq \left(Y(0) - \frac{\gamma S^0 d_s}{d_{min}} e^{-d_{min} t}\right) + \frac{\gamma S^0 d_s}{d_{min}}$$

This implies that for any initial condition in Ω_5,

$$\begin{aligned} \limsup_{t\to\infty} Y(t) &\leq \limsup_{t\to\infty} \left(\left(Y(0) - \frac{\gamma S^0 d_s}{d_{min}} e^{-d_{min} t}\right) + \frac{\gamma S^0 d_s}{d_{min}} \right) \\ &\leq \frac{\gamma S^0 d_s}{d_{min}} \end{aligned}$$

From the second equation from the model (7),

$$A'(t) \leq d_A(A_0(t) - A)$$

The equation takes the form

$$A \leq \frac{M e^{d_A t}}{e^{d_A t}}$$

where $M = \max_t |A_0(t)|$. Finally

$$\begin{aligned} \limsup_{t\to\infty} A(t) &\leq \limsup_{t\to\infty} \frac{M e^{d_A t}}{e^{d_A t}} \\ &\leq M \end{aligned}$$

This implies that system (3) is ultimately uniformly bounded in forward time.

A.2 Proof of Theorem 3.2

The local stability of the sterile steady state $E_0(t)$ can be determined by the Floquet exponents of the variational equation corresponding to (3)and is given as

$$M' = \begin{pmatrix} -d_s & 0 & 0 & -\frac{1}{\gamma}G_1(S^0) & -\frac{1}{\gamma}G_2(S^0) \\ 0 & -d_A & 0 & f(A^*) & -f(A^*) \\ 0 & 0 & -d_x & 0 & K_2(S^0, A^*) \\ 0 & 0 & 0 & p43 & 0 \\ 0 & 0 & 0 & 0 & p55 \end{pmatrix} M \tag{10}$$

where

$$p43 = G_1(S^0) - d_u - K_1(S^0, A^*)$$
$$p55 = G_2(S^0) - d_+ - K_2(S^0, A^*)$$

The corresponding fundamental matrix $\Phi(t)$ is obtained as

$$\Phi(t) = \begin{pmatrix} e^{-d_s t} & 0 & 0 & \cdot & \cdot \\ 0 & e^{-d_A t} & 0 & \cdot & \cdot \\ 0 & 0 & e^{-d_x t} & 0 & \cdot \\ 0 & 0 & 0 & e^{\int_0^t [G_1(S^0) - d_u - K_1(S^0, A^*(s))]ds} & 0 \\ 0 & 0 & 0 & 0 & e^{\int_0^t [G_2(S^0) - d_+ - K_2(S^0, A^*(s))]ds} \end{pmatrix}$$

Next, evaluating the fundamental solution at $t = T$ yields the multipliers $e^{-dsT}, e^{-daT}, e^{-dxT}$ and $e^{T[G_1(S^0) - d_u - [K_1(S^0, A^*(t))]_m]}, e^{T[G_2(S^0) - d_+ - [K_2(S^0, A^*(s))]_m]}$ where

$$[K_1(S^0, A^*(t))]_m = \frac{1}{T}\int_0^T K_1(S^0, A(s))ds$$

where K_1 is a continuous T periodic function. Then it follows immediately that

$$\lambda_1 = -d_s$$
$$\lambda_2 = -d_A$$
$$\lambda_3 = -d_x$$
$$\lambda_4 = G_1(S^0) - d_u - [K_1(S^0, A^*(t))]_m$$
$$\lambda_5 = G_2(S^0) - d_+ - [K_2(S^0, A^*(t))]_m$$

The first three eigenvalues λ_1, λ_2 and λ_3 are negative and $\lambda_4 < 0$ if $G_1(S^0) - d_u - [K_1(S^0, A^*(t))]_m < 0$ and $\lambda_4 < 0$ if $G_2(S^0) - d_+ - [K_2(S^0, A^*(t))]_m < 0$. This implies that $E_0(t)$ is locally asymptotically stable if λ_4, λ_5 both are negative. Since $G_1(S^0) - d_u - K_1(S^0, A_0) < 0$ and $G_2(S^0) - d_+ - K_2(S^0, A_0) < 0$. Choose an $\epsilon > 0$ small enough such that

$$G_1(S^0 + \epsilon) - d_u - K_1(S^0 + \epsilon, A_0 + \epsilon) < 0$$
$$G_2(S^0 + \epsilon) - d_+ - K_2(S^0 + \epsilon, A_0 + \epsilon) < 0$$

Since $S'(t) \le d_s(S^0 - S)$ and $A'(t) \le d_A(A_0 - A)$, we conclude that for large $t > 0$, $S(t) \le S^0 + \epsilon$ and $A(t) \le A_0 + \epsilon$. From the u, v equation of model (3)

$$\begin{aligned}
u'(t) + v'(t) &= \Big(G_1(S) - d_u - K_1(S,A)\Big)u - k_3 x u + \Big(G_2(S) - d_+ - K_2(S,A)\Big)v - k_4 x u \\
&\le \Big(G_1(S) - d_u - K_1(S,A)\Big)u + \Big(G_2(S) - d_+ - K_2(S,A)\Big)v \\
&\le \Big(G_1(S^0+\epsilon) - d_u - K_1(S^0+\epsilon, A_0+\epsilon)\Big)u \\
&\quad + \Big(G_2(S^0+\epsilon) - d_+ - K_2(S^0+\epsilon, A_0+\epsilon)\Big)v \\
&\le \eta(u+v)
\end{aligned}$$

where
$\eta = \min\Big\{G_1(S^0+\epsilon) - d_u - K_1(S^0+\epsilon, A_0+\epsilon), G_2(S^0+\epsilon) - d_+ - K_2(S^0+\epsilon, A_0+\epsilon)\Big\}$.
This implies that $\limsup_{t\to\infty} u(t) + v(t) \le 0$. Using the positivity of the u and v, we conclude that $\lim_{t\to\infty} u(t) = 0$ and $\lim_{t\to\infty} v(t) = 0$. Next, $x'(t) \le K_2(S,A)v$. This implies that

$$x'(t) \le K_2(S^0+\epsilon, A_0+\epsilon)v$$

This leads to

$$\begin{aligned}
x(t) &\le K_2(S^0+\epsilon, A_0+\epsilon)\int v(t)dt \\
\limsup_{t\to\infty} x(t) &\le K_2(S^0+\epsilon, A_0+\epsilon)\limsup_{t\to\infty}\int v(t)dt \\
&\le \eta_2 \int \limsup_{t\to\infty} v(t)dt \\
\limsup_{t\to\infty} x(t) &\le \epsilon'
\end{aligned}$$

where $\epsilon' = \eta_2\epsilon_1$ and for $t > T$ large enough and for every $\epsilon_1 > 0$, $\limsup_{t\to\infty} v(t) < \epsilon_1$. From the positivity of the x, we conclude that $\lim_{t\to\infty} x(t) = 0$.
