# Supplementary material for: Effective antibiotic dosing in the presence of resistant strains
Source: PLoS One. 2022 Oct 10;17(10):e0275762. doi: 10.1371/journal.pone.0275762 (PMC9551627; doi:10.1371/journal.pone.0275762)
Supplement: Attachment — Submitted filename: Response to Reviewers Comments.docx [file pone.0275762.s002.docx]

We would like to thank the reviewers for the speedy turnaround and valuable comments. Their feedback has allowed us to improve the manuscript. We have updated the manuscript in light of the comments and a point by point response is given below.

**Editorial Comments:**

**1.** Please ensure that your manuscript meets PLOS ONE's style requirements, including those for file naming.

**Response:** The manuscript has been converted to the PLOS ONE format meeting the style requirements.

**2.** Please update your submission to use the PLOS LaTeX template. The template and more information on our requirements for LaTeX submissions can be found at http://journals.plos.org/plosone/s/latex.

**Response:** We have updated the submission accordingly.

3. In your Data Availability statement, you have not specified where the minimal data set underlying the results described in your manuscript can be found.

**Response:** All data used in our simulation is available in the manuscript.

**Reviewer #1:**

If possible can the authors provide some pilot study data using the model DGDM. Please justify how this model will be better than already available systems.

**Response:** In section 2 we provide a benchmark for the method by applying it to a simple well studied model. The two main contributions of the study are proposing a model for HGT via transformation and determining a discrete optimal dosing schedule. While there are many models in the literature that study HGT via conjugation, there are only a few models that look into HGT via transformation, further we have not seen any work on optimizing antibiotic dosing in this case. We also would like to note that in the case of our benchmarking model, the DGDM allows us to easily determine a discrete control (dosing regimen) for a continuous system, standard methods such as the Pontryagin’s Maximum Principle need significant modification to be applicable in such cases.

**Reviewer #2:**

**1)** Kindly, rewrite the abstract, including its all contents, that introduction to literature review, study gaps, methodology, results, discussion, conclusion and future prospects, briefly in one paragraph.

**Response:** The abstract has been updated in light of the comment.

**2)** Kindly arrange in-text references as per journal format.
**Response:** The manuscript has been updated in light of the comment.

**3)** Kindly give the line numbers as per journal format. **Response:** The manuscript has been updated in light of the comment.

**4) Kindly provide with lab-work pictures.
Response:** Since this is a modeling and simulation study, the pictures are those obtained from simulation. We are planning a follow up work with a biologist to verify our model with in vitro experiments.

**5)** Arrange the references as per journal format.
**Response:** The manuscript has been updated in light of the comment.

**6)** Kindly include conclusion section.
**Response:** The discussion section (5) was envisaged to be a conclusion section, the section title has been changed and it has also been updated.

**7)** Add future prospects of this study.
**Response:** The conclusion section (6) has been updated with a discussion of future directions.

**8)** Kindly mention the methodology and result section separately.
**Response:** Each of the sections where models are presented and discussed, are already organized in this manner. Since we present three different models, one of which has been formulated and studied in this work while we use the other two are used to present the optimal dosing strategy, it would be difficult to provide the methodology and results in two sections. As mentioned each of sections 3, 4 and 5 are already informally organized in this manner, where we present the methodology and then discuss the results.

**9)** Kindly elaborate the literature review along with references.

**Response:** The introduction section has been updated, with more discussion of the models in the literature, in light of the comment.

**10)** Are the study gaps really come up with the need of this investigation. Kindly elaborate this point.
**Response:** This point has been addressed in the introduction, there are few modeling studies of antibiotic resistance acquired via transformation, and moreover to our knowledge no study has been done to look into optimal dosing treatments in this case. Our study is an effort in this direction.

**11)** Kindly include the models in literature used to carry out this study or those which are just mentioned in discussion section. It is recommended to briefly discuss each model from literature with your study models.

**Response:** The introduction and conclusions sections have been updated, reference to the models mentioned in the conclusion have been added and more discussion of the models in the literature mentioned in the introduction has been added, in light of the comment.

**12)** it is suggested to make a table in manuscript regarding the parameters of this study, instead of using them in appendix text.

**Response:** The manuscript has been updated in light of the comment.
