# Supplementary material for: Effective antibiotic dosing in the presence of resistant strains
Source: PLoS One. 2022 Oct 10;17(10):e0275762. doi: 10.1371/journal.pone.0275762 (PMC9551627; doi:10.1371/journal.pone.0275762)
Supplement: Attachment — Submitted filename: Response to Reviewers Comments.docx [file pone.0275762.s003.docx]

We would like to thank the reviewers for the speedy turnaround and valuable comments. It seems like we may not have fully comprehended reviewer#1’s concern in our earlier response. A more detailed and hopefully pertinent response is given below.

**Reviewer #1:** Authors have responded to the comment but have not given any satisfactory answer to the comment. Since there is almost no work on optimizing antibiotic dosing, wet lab experiments must be generated to verify the model. The validity of the model must be associated with realtime data. it seems to be a hypothetical model.

**Response:** Our model is built upon and extends previous work. Although there is not a great deal of literature on HGT via transformation, however, there are studies where models are presented. For example, [1] and [2] have proposed models for HGT via transformation. Our work extends the existing work by incorporating a compartment for cell-free DNA in the model, which we hope better captures the dynamics of the process. Moreover, we have set our model in a chemostat setting, as we want as a proxy for a more complex organism.

Further, we have presented some rigorous mathematical analyses of our model using Floquet theory. We show that the system is bi-stable under a constant periodic dosing regimen. This means that the same periodic dose may or may not be successful for different initial bacterial populations. This provides the rationale to look for optimal dosing regimens.

We also would like to summarize some prior studies on optimal antibiotic dosing. Most of these are based on proposing a model for bacterial growth, including mechanisms for resistance acquisition and then searching (using different algorithms) for the optimal dosing, which results in bacterial eradication with low total antibiotic dosages. In [3], the authors propose an ODE-based model and its stochastic analog for Horizontal Gene Transfer (without specifying the exact mechanism) and then use genetic algorithms to search for the optimal dosing regimen. In [4], the authors study optimal antibiotic dosing strategies using an ODE-based model for bacterial growth in a chemostat setting and optimal control theory. In [5], a hybrid DE and Agent-based model is considered for TB, and the authors also use genetic algorithms to determine the optimal antibiotic dosing schedule. All these works are essentially modeling and simulation efforts (of course, the dynamics of bacterial growth and resistance acquisition (if considered) must be grounded in biology.

Moreover, the effects of the antibiotic need to be modeled by a proper pharmacodynamic function, which has been the case in all these studies as well as our work which is under consideration). They can provide a framework for wet lab experiments, as has been suggested by the reviewer. For example, in [6], the authors have actually used the framework presented in [3] for their wet lab experiments.

We have also updated the section where we present our model in light of the comment. Further, the table of parameters has been updated as well. We hope that our response will be able to address the reviewer's concern.

**References:**

[1] Lu N, Massoudieh A, Liang X, Kamai T, Zilles JL, Nguyen TH, et al. A kinetic model of gene transfer via natural transformation of Azotobacter vinelandii. Environmental Science: Water Research & Technology. 2015;1(3):363–374.

[2] Johnsen PJ, Dubnau D, Levin BR. Episodic selection and the maintenance of competence and natural transformation in Bacillus subtilis. Genetics. 2009;181(4):1521–1533

[3] Paterson IK, Hoyle A, Ochoa G, Baker-Austin C, Taylor NGH. Optimising Antibiotic Usage to Treat Bacterial Infections. Scientific Reports. 2016;6(1):37853

[4] Peña-Miller R, Lähnemann D, Schulenburg H, Ackermann M, Beardmore R. Selecting Against Antibiotic-Resistant Pathogens: Optimal Treatments in the Presence of Commensal Bacteria. Bulletin of Mathematical Biology. 2012;74(4):908–934

[5] Cicchese, J.M., Pienaar, E., Kirschner, D.E. *et al.* Applying Optimization Algorithms to Tuberculosis Antibiotic Treatment Regimens. *Cel. Mol. Bioeng.* **10**, 523–535 (2017)

[6] Hoyle A, Cairns D, Paterson I, McMillan S, Ochoa G, Desbois AP. Optimising eﬀicacy of antibiotics against systemic infection by varying dosage quantities and times. PLOS Computational Biology. 2020;16(8):1–20
